# Influence of Chia Seed, Buckwheat and Chestnut Flour Addition on the Overall Quality and Shelf Life of the Gluten-Free Biscuits

**DOI:** 10.17113/ftb.59.04.21.7204

**Published:** 2021-12

**Authors:** Gonul Silav-Tuzlu, Zeynep Tacer-Caba

**Affiliations:** 1 Istanbul Aydin University, Department of Food Engineering, İnönü Caddesi No:38, 34295 Florya, Istanbul, Turkey; 2 Bahcesehir University, Department of Gastronomy and Culinary Arts, Ihlamur Yildiz Caddesi No. 8 Gayrettepe, 34353 Besiktas, Istanbul, Turkey

**Keywords:** buckwheat, chestnut, chia, gluten-free biscuits, sensory characteristics, coeliac disease, nutritional value

## Abstract

**Research background:**

In spite of being a significantly growing segment, there are still problems regarding the nutritional, technological and sensory profiles of gluten-free products. Thus, the combination of a variety of functional ingredients is required in order to achieve the desired product quality.

**Experimental approach:**

Three types of flour, chestnut, buckwheat and potato, were chosen in this study because they are all gluten-free, nutritionally richer and technologically more advantageous than wheat flour. They are combined with chia seeds, which are also functional ingredients as they are rich in dietary fibre and unsaturated fatty acids. Therefore, this study aims to evaluate the utilization of chia seeds with chestnut, buckwheat and potato flour in biscuits as overall quality enhancers in gluten-free products. The proximate composition, total phenolic content, antioxidant activity, some biscuit quality parameters and the sensory properties of the samples were investigated, and some changes in these products during storage were monitored and evaluated.

**Results and conclusions:**

According to the results, biscuits with chestnut flour had the highest phenolic content (400.2 mg gallic acid equivalent (GAE) per 100 g dry sample) and total antioxidant activity (155.5 mg Trolox equivalent (TE) per 100 g dry sample). Biscuits with chestnut and chia seeds had the hardness of 30.1 N (p<0.05). In addition, the use of chia seeds significantly increased the overall acceptability and flavour scores according to the sensory analysis results. During storage, chia seeds affected the oxidation stability; however, the fatty acid profile remained almost unchanged, except for the losses in lauric, stearic and α-linolenic acids (p<0.05). In conclusion, the biscuits with chestnut and chia seeds were more attractive than those made with other types of flour, with remarkably better nutritional characteristics and sensory attributes.

**Novelty and scientific contribution:**

The study fulfils a need for the growing gluten-free market by combining together the functional nutrients of chia seeds, chestnut flour and buckwheat flour to achieve the nutritionally improved and organoleptically acceptable gluten-free biscuits. Furthermore, this study makes an overall evaluation of the changes in product quality during storage to provide new ideas for an overall innovation in the gluten-free food market.

## INTRODUCTION

Coeliac disease is defined as the abnormal response of the immune system to wheat gluten and related prolamins of rye and barley (*1*). It mainly affects the small intestine as inflammation and causes a decrease in the absorption of some nutrients. It is medically defined and considered as being among the most common life-long disorders and a gluten-free diet is the recommended therapy for those patients (*1*, *2*). Another group, perceived gluten sensitivity, also necessitates a gluten-free diet. Recently, the number of people suffering from chronic diseases has increased as a result of changing lifestyles. Accordingly, raising consumer awareness of the relationship between food and health has become the centre of attention over the years. Thus, myriad of consumers in addition to the coeliac patients prefer to consume gluten-free products to sustain a healthier lifestyle (*3*). Therefore, gluten-free food consumption has recently become an increasing trend for mainly these three different types of consumers with varying expectations (*4*). The current global market size is around 3126 million US dollars and is expected to reach US$5279 million by 2022 with a steady growth (*5*). The growth may be explained by the increase in the number of coeliac disease patients and diagnosed cases of wheat allergy and gluten sensitivity. However, from a sensorial and nutritional perspective, the gluten-free products are still a bit far away from meeting the demand of most consumers (*2*). Therefore, there is still a need for further research in order to better understand consumer expectations of gluten-free products.

Gluten is technologically crucial in sustaining the viscoelastic properties of bakery products; therefore, it is challenging to produce high quality gluten-free products acceptable to consumers. Consequently, research has focused on numerous types of ingredients with non-gluten protein sources to enhance the overall quality in technological, sensorial, functional and nutritional aspects (*1*, *6*, *7*).

Nutritional properties of gluten-free products should be more in focus, as traditional gluten-free ingredients/flour are high in carbohydrates but low in proteins and antioxidants. Therefore, the use of dietary fibre-rich ingredients is proposed to improve the nutritional quality of these products (*8*, *9*). Previous reports highlighted the higher propensity of coeliac patients for excessive consumption of fat- and sugar-containing foods. This inclination could be a contributing factor to compensate for the limitations of gluten-free diet (*4*). Therefore, sustaining the nutritional profile of gluten-free products remains a serious challenge, alongside their technical properties. Biscuits have significant attributes such as relatively long shelf-life, convenience and good eating quality and suitability for different nutritional/functional novelty trials, which makes them good alternatives for gluten-free trials (*10*). Therefore, in this study, potato (*Solanum tuberosum*) flour with buckwheat (*Fagopyrum esculentum*) flour and chestnut (*Castanea sativa* Mill.) flour were used together with chia (*Salvia hispanica* L.) (Lamiaceae) seeds in gluten-free biscuit formulations to enhance both nutritional and technological drawbacks.

Potato (*Solanum tuberosum*) flour is commonly used in gluten-free products because it is rich in essential amino acids (especially lysine), protein dietary fibre and it contains several phytochemicals (phenolics, flavonoids and carotenoids) in addition to its high carbohydrate content (*11*, *12*).

Buckwheat (*Fagopyrum esculentum*) is a pseudocereal. Buckwheat flour, which is rich in catechins serving as antioxidants, also includes specific amino acids (such as lysine, histidine, valine and leucine), as well as minerals. Buckwheat proteins are mainly composed of albumins and globulins, with only a low amount of prolamins. In this respect, they are similar to leguminous proteins and considered as gluten-free (*13*). The dietary fibre is of great significance and, together with the key polyphenols and potential antioxidant activities in buckwheat, adds functional properties that are beneficial for health (*10*, *14*).

Chestnut (*Castanea sativa* M.) flour is popular in gluten-free formulations, due to its high protein quality with essential amino acids, high dietary fibre content and vitamins (such as vitamin E, and vitamin B group) and minerals like potassium, phosphorous and magnesium. Recent studies have evaluated the effects of supplementation of gluten-free biscuits and bread with different levels of chestnut flour (*2*, *15*).

The chia plant (*Salvia hispanica* L.) originates from Central and South America (mainly southern Mexico and northern Guatemala), and particularly its seeds possess a technically unique property by means of which they can absorb large amounts of water to immediately form a transparent gel referred to as ’chia mucilage‘ (*16*, *17*). This allows it to be used as a thickener, gel former, chelator, or fat replacer in different applications of the food industry such as bakery products. Chia seeds are nutritionally significant sources as they accommodate high levels of dietary fibre, protein and oil (particularly rich in polyunsaturated fatty acids (omega-3 fatty acids (α-linolenic acid, 54–67%) and omega-6 (linoleic acid, 12–21%)) and are low in saturated fatty acids. They also possess other significant components such as tocopherols and phenolic compounds (chlorogenic acid, caffeic acid, myricetin, quercetin and kaempferol) (*18*).

Although some improvements have been achieved, and despite their technical properties, the nutritional profile of gluten-free products still poses serious challenges. In this context, this study aims to evaluate the nutritional, technological and sensorial profile of gluten-free biscuits and to monitor the product quality and changes during storage in order to develop an overall understanding of the products.

## MATERIALS AND METHODS

### Materials

Four different formulations of dough prepared with: (*i*) buckwheat flour, (*ii*) chestnut flour, (*iii*) buckwheat flour and chia seeds, and (*iv*) chestnut flour and chia seeds were used for the production of gluten-free biscuits. For these formulations, buckwheat flour (Ecology Market Food Industry Trade Co., Istanbul, Turkey), chestnut flour (Naturelka CC Tourism Co., Aydın, Turkey), potato flour (Sakarya Agricultural Products, Sakarya Turkey), chia seeds (Güzel Ada Food, Istanbul, Turkey) lactose-free milk (Pınar Dairy, Izmir, Turkey) were provided by the respective companies. In addition, margarine, sugar and whole eggs were purchased from a local grocery store in Turkey.

### Reagents

Ethanol (≥99.8%), acetone (≥99%) and copper were from Delta Agricultural Chemicals Industry and Trade Inc. (Antalya, Turkey), sulfuric acid, sodium hydride, petroleum ether, hydrochloric acid, boric acid, potassium chloride, sodium bicarbonate, aluminium chloride and trifluoroacetic acid were from Merck (Darmstadt, Germany). Gallic acid, 2,2-diphenyl-1-picrylhydrazyl (DPPH) and Trolox ((±)-6-hydroxy-2,5,7,8-tetramethylchromane-2-carboxylic acid) used in this work were from Sigma-Aldrich Co. (Merck, St. Louis, MO, USA).

### Biscuit production

In this study, four different types of gluten-free biscuit formulations were produced according to a modified method by Öksüz and Karakaş (*19*). The details are depicted in Table 1. The ingredients were mixed to form the dough and rolled to a thickness of 0.1 cm. Then the biscuits were moulded with a cutter of 50 mm diameter and baked in a conventional oven at 150 ºC for about 25 min. Afterwards, they were left to cool down to room temperature for about 1 h and then kept in airtight containers.

**Table 1:** Biscuit formulations

Ingredient	Biscuit with buckwheat flour	Biscuit with chestnut flour	Biscuit with buckwheat flour and chia seeds	Biscuit with chestnut flour and chia seeds
*m*(buckwheat flour)/g	75	-	75	-
*m*(chestnut flour)/g	-	75	-	75
*m*(potato flour)/g	75	75	75	75
*m*(chia seeds)/g	-	-	15	15
*m*(sugar)/g	55	55	55	55
*V*(lactose-free milk)/mL	40	40	45	45
*m*(margarine)/g	45	45	45	45
*V*(eggs)/mL	6	6	6	6
*m*(sodium bicarbonate)/g	3	3	3	3

### Proximate analyses

Total moisture, ash, protein and fat contents were determined according to the AOAC methods. The total moisture content was determined by measuring the mass loss after drying for 1 h at 130 ºC according to the AOAC method 925.10 (gravimetric air oven method) (*20*). The total ash content was determined according to the gravimetric AOAC method 923.03 by ashing the samples in the muffle furnace for 16 h at 600 ºC (*21*). Total protein content of the samples was measured according to Kjeldahl method (AOAC 920.87) with the factor of 5.7 for converting the total nitrogen into total protein (*22*). Soxhlet (gravimetric) method (AOAC 945.16) was used to extract the oil from the samples and measure its content (*23*).

The total amount of dietary fibre was determined using the Total Dietary Fiber Assay Kit (TDF100A; Sigma-Aldrich, Merck), based on AOAC method 985.29 (enzymatic-gravimetric method) (*24*). The total content of carbohydrates was calculated by subtracting the total amounts of moisture, protein, ash and fat constituents of the sample from 100. The obtained value presents the carbohydrate content of the sample (g/100 g). The total energy (kcal/100 g) was calculated using the conversion factors 9 for each g of fats, and 4 for each g of carbohydrates and proteins.

### Determination of total phenolic content and antioxidant activity of biscuits

A slightly modified extraction procedure was applied to prepare the biscuit extracts for measuring the total phenolic content and antioxidant activity (*25*). In summary, the samples (2 g) were first extracted by adding 15 mL of 80% methanol and then shaken on an orbital shaker (Model SSL1; Cole-Parmer, Staffordshire, UK) for 15 min (4000×*g*), sonicated (model PMUY-4L-D; Protech Mechanical Equipment Co, Ltd., Zhengzhou, PR China) for 15 min and centrifuged (model Rotofix 32A; Hettich, Tuttlingen, Germany) at 4000×*g* for 20 min. The extraction was repeated until 30 mL of extract were collected in two replicates for each sample.

#### Total phenolics

Total phenolic content of biscuits was measured using the Folin-Ciocalteu method (*25*). According to the method, 750 μL of diluted Folin-Ciocalteu solution (1:10) were mixed with 100 μL of biscuit extract and 750 μL of sodium carbonate solution (6%). After incubation for 90 min in the dark, the absorbance of the solution was measured at 750 nm against water with a spectrophotometer (V-1800; Shimadzu, Kyoto, Japan). The analysis was repeated twice for each sample. Gallic acid was used as the reference and the results are given as gallic acid equivalent (GAE) per 100 g sample.

#### Total antioxidant activity

The total antioxidant activity (TAA) of the biscuit extracts was measured using the DPPH radical scavenging activity method (*26*). A volume of 1 mL of biscuit extract was added to a test tube containing 4 mL of methanol (80%) and 1 mL of freshly prepared DPPH solution (1 mmol/L), and the final concentration of DPPH solution was adjusted to 0.167 mmol/L. Then the tubes were kept in the dark for 30 min and sample absorbance was measured at 517 nm with spectrophotometer (V-1800; Shimadzu Corp., Tokyo, Japan), using Trolox as the reference.

### Biscuit quality evaluation

The diameter and thickness of the biscuit samples were measured using a digital caliper. Bulk density was measured by dividing the mass of each sample by its volume. The spread ratio was measured by dividing the diameter (D) by the thickness (T). All measurements were made in triplicates. The volume of the biscuits was measured using the rapeseed displacement method by comparing the change in the known volume of rapeseed with each biscuit sample, as described in AACC method 10-05.01 (*27*) and water absorption test was performed according to the AACC method 56-30.01 (*28*). In the latter, ground samples (0.2 g) were weighed into pre-weighed centrifuge tubes and 10 mL of distilled water were added. The tubes were kept in a water bath at 30 °C for 30 min, vortexed for 5 s every 5 min and centrifuged at 3000×*g* (Hettich Rotofix 32A; Merck) for 10 min. The percentage of absorbed water was measured by weighing the sediment.

The colour of the biscuit samples was measured using the colour chromameter (CR-400; Konica Minolta Inc., Tokyo, Japan), using the Hunter scale of L*, a*, b*. Six samples from each biscuit formulation were measured and the average was calculated. Colour parameters for the standard white plane were as follows: *L*_0_=95.9, *a*_0_=0.2 and *b*_0_=2.3.

The difference between the colour parameters of darkness/lightness (L* values), redness (a* values) and yellowness (b* values) of the biscuit samples and the standard white plane parameters given above were calculated to measure the total colour change (Δ*E*) according to the equation below:



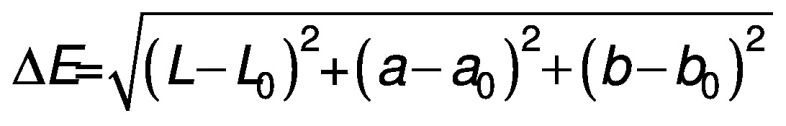



Hardness was measured using TA.XTplus Texture Analyzer (Stable Micro Systems, Surrey, UK), fitted with a 50-kg load cell and coupled with Texture Expert Software, v. 1.22 (*29*). Measurements were made approx. 24 h after baking to measure the biscuit fracture, and each biscuit was compressed by a three-point bending rig (HDP/3PB) and blade (90 mm long and 3 mm thick) at a crosshead speed of 3 mm/s. The biscuits were 10 mm thick. The maximum force (N) measured in the force-time (distance) curve was taken as the value of hardness (*30*).

### Sensory evaluation of the biscuits

Sensory properties of biscuit samples were evaluated by 10 panellists. The panellists were females, aged between 25 and 35 and they were semi-trained to evaluate and compare the samples for parameters of appearance, colour, smell, taste texture/mouth feel and overall acceptance, on a 9-point hedonic scale ranging from 1 for dislike extremely to 9 for like extremely, according to a modified method of Moretti *et al.* (*31*). In the preparatory session, the panel members had a thorough discussion in order to clarify each attribute before their evaluation. For each parameter, the average of the relevant scores given by the panellists was calculated.

### Changes detected during the storage of biscuits

All biscuit samples were stored at room temperature (around 22-23 ºC) in locked sterile bags for a period of 45 days. Moisture content of the samples was measured as described above, peroxide value (PV) was determined by iodometric method ISO 3960:2017 (*32*). In addition, total acidity was determined by measuring the total amount of treatable acids. Their pH values were measured on the 1st, 20th and 45th day. Fatty acid composition of the freshly baked biscuit samples and the samples that were stored for 6 months was evaluated with gas chromatography-flame ionization detector (GC–FID) model CLARUS 580 (Perkin Elmer, Shelton, CT, USA) and a BPx70 column (30 m, 0.25 mm, 0.25 mm film thickness) (Trajan Scientific and Medical, Ringwood, VA, Australia). The fatty acid content of the biscuits was expressed as the percentage of total oil content.

### Statistical analysis

The data were analysed using the SPSS software, v. 18 (*33*). Analysis of variance (ANOVA) was used for comparison. The Duncan’s test was used as the *post-hoc* test to determine the differences. Differences between samples were calculated at 95% significance level (*34*).

## RESULTS AND DISCUSSION

Sustaining the nutritional properties of gluten-free products is among the most recent interests for the researchers, as traditional gluten-free ingredients/flour are low in proteins and antioxidants but only rich in carbohydrates (*8*).

### Proximate composition of raw materials

According to the findings presented in Table 2, raw materials had significant differences (p<0.05) in terms of proximate components. Buckwheat flour had significantly higher amount of protein (11.4%) than the chestnut and potato flour (6.7 and 5.2% respectively), but lower (p<0.05) than that of the chia seeds (23.1%). Mass fractions of ash (1.88%), fat (1.7%) and carbohydrates (72.4%) in buckwheat flour were not significantly different than in the other flour sources (p>0.05). Similar to our findings, the moisture, protein, ash and fat mass fractions of buckwheat flour that was used as the raw material for cookies with rice flour were 11.3, 12.3, 2.2 and 2.9%, respectively in a study by Torbica *et al.* (*35*). In another study, buckwheat flour carbohydrate mass fraction was reported to be 69.7% (*36*).

**Table 2:** Proximate analyses of raw materials

Raw material	*w*(protein)/ %	*w*(dietary fibre)/%	*w*(fat)/%	*w*(ash)/%	*w*(CHO)/%	*w*(moisture) /%	*E/* (kcal/100 g)
Buckwheat flour	(11.4± 0.2)^b^	(7.6± 0.3)^c^	(1.7± 0.1)^b^	(1.0± 0.2)^b^	(72.4± 0.3)^a^	(12.9± 0.1)^b^	(320.0± 0.3)^b^
Potato flour	(6.7± 0.2)^c^	(6.4± 0.5)^c^	(3.0± 0.1)^ab^	(2.8± 0.2)^ab^	(81.1± 0.4)^a^	(6.4± 0.2)^c^	(353.1± 0.5)^ab^
Chestnut flour	(5.2± 0.1)^c^	(18.0± 0.2)^b^	(1.6± 0.0)^b^	(3.83± 0.09)^a^	(68.4± 0.2)^a^	(21.0± 0.1)^a^	(236.5± 0.3)^c^
Chia seeds	(23.1 ±0.1)^a^	(34.4± 0.4)^a^	(29.3± 0.0)^a^	(4.58± 0.04)^a^	(36.2± 0.1)^b^	(6.8± 0.1)^c^	(363.3± 0.2)^a^

*E*=total energy, CHO=carbohydrates. The results are shown as mean value±standard deviation of duplicate analyses. Values in the same column with different letters differ significantly (p<0.05)

Chestnut flour had significantly lower (p<0.05) protein mass fraction (5.2%) than the other ingredients. This finding is commensurate with previous findings reported in the literature, since chestnut flour reported by Šoronja‐Simović *et al.* (*37*) and Lopes *et al.* (*38*) contained 5.3 and 5.7% protein, respectively. Its fat and carbohydrate mass fractions were statistically different from buckwheat flour (p>0.05). As expected, buckwheat flour and chestnut flour also had higher mass fractions of total carbohydrates than chia seeds (p<0.05). Chestnut flour had a lower moisture (7.7%) and ash (2.1%), but similar protein (5.7%), fat (1.6%), dietary fibre (20.7%) and carbohydrate (62.3%) mass fractions in an earlier study (*38*).

Total mass fractions of protein (6.7%), ash (2.8%) and carbohydrates (81.1%) in potato flour were slightly different with respect to the other flour sources; although the fat mass fraction was significantly higher than the other ingredients (except for the chia seeds) and the moisture mass fraction was lower (p<0.05). In a recent study (*11*), potato flour was also used as a raw material for another gluten-free product and reported to contain 9.17% protein, 3.86% ash, 0.26% fat and 20.51% dietary fibre. Therefore, proximate mass fractions were generally higher than in the potato flour used in this study. In contrast, the potato flour used in another study (*39*) was reported to have 5.47% protein, 2.90% ash, 79.83% total carbohydrate and 5.29% dietary fibre, thus being more similar to the raw material presented in this study.

Chia seeds had significantly higher protein (23.1%), ash (4.58%) and dietary fibre (34.4%), but lower carbohydrate (36.2%) content than the flour sources (p<0.05) used in this study. Comparable to the current findings, chia seeds were reported to have 9.3% moisture, 25.1% protein, 5.5% ash and 26.2% fat (*40*). Total dietary fibre in chia seeds and chia seed flour was indicated as quite similar to (around 37.4%) (*41*), or slightly lower (29.6 and 30.1%) (*42*), respectively than our findings (34.4%).

### Proximate composition of biscuit samples

According to the results of the proximate component analyses of biscuit samples (Table 3), the compositional differences in raw materials affected the proximate compositions of the biscuits, as expected (p<0.05). Protein mass fraction in different biscuit samples was significantly different (p<0.05). The biscuits baked with chestnut flour had the lowest mass fraction of protein on dry mass basis (4.8%), which is in accordance with the relatively lower protein mass fraction of the chestnut flour. Biscuits with buckwheat and chia had the highest mass fraction of proteins (8.2%). Buckwheat flour had significantly higher mass fraction of protein (11.4%; Table 2) than the chestnut and potato flour, although it was lower than that of the chia seeds. Therefore, incorporation of buckwheat flour in gluten-free formulations was found to be an effective method for increasing their protein mass fraction. Similar results have been reported in a study using wholegrain buckwheat flour in gluten-free crackers (*43*). The increase in total protein mass fraction with chia seed flour was also similar to other studies, as the addition of 5 to 20% chia seed flour in the study by Kaur *et al.* (*36*) resulted in increasing mass fractions of measured protein in biscuit samples, ranging from 9.7 to 11.7%.

**Table 3:** Proximate components, total phenolics, antioxidant activity and quality parameters of biscuits

Sample	*w*(protein)/ %	*w*(ash)/ %	*w*(moisture)/ %	*w*(fat)/ %	*w*(dietary fibre)/%	*E/* (kcal/ 100 g)	*w*(total phenolics as GAE)/(mg/100 g)	TAA as TE/(mg/100 mg)	*d*/mm	*h*/mm	*m*/g	*v*/(cm^3^/g)	Spread ratio	WA/g	Hardness/N
Biscuit with buckwheat	(7.1± 0.0)^b^	(3.7± 0.4)^a^	(5.2± 0.3)^a^	(17.6± 0.0)^b^	(11.5± 0.0)^d^	(406.7± 0.3)^a^	(387.0± 9.1)^a^	(132.2± 5.9)^b^	(60.0± 0.9)^a^	(11.1± 0.4)^a^	(20.2± 1.3)^a^	(1.25± 0.03)^a^	(5.4± 0.0)^a^	(2.6± 0.0)^b^	(48.7± 3.7)^a^
Biscuit with chestnut	(4.8± 0.4)^d^	(3.7± 0.6)^a^	(5.9± 0.3)^a^	(17.7± 0.0)^b^	(13.2± 0.0)^c^	(397.5± 2.3)^ab^	(400.2± 3.7)^a^	(155.5± 4.4)^a^	(58.1± 2.7)^a^	(11.4± 0.8)^a^	(20.8± 2.9)^a^	(1.24± 0.22)^a^	(5.1± 0.1)^a^	(2.5± 0.1)^b^	(38.3± 5.7)^b^
Biscuit with buckwheat and chia	(8.2± 0.1)^a^	(3.7± 0.6)^a^	(5.4± 0.8)^a^	(18.8± 0.1)^a^	(17.2± 0.1)^b^	(388.6± 0.5)^b^	(248.2± 7.9)^c^	(78.2± 2.3)^d^	(60.2± 1.3)^a^	(10.7± 1.1)^a^	(22.0± 2.3)^a^	(1.14± 0.01)^b^	(5.6± 0.7)^a^	(3.3± 0.2)^a^	(37.6± 8.1)^b^
Biscuit with chestnut and chia	(5.7± 0.2)^c^	(3.8± 0.6)^a^	(5.5± 0.5)^a^	(18.3± 0.4)^ab^	(19.3± 0.1)^a^	(376.8± 4.2)^c^	(298.5± 3.3)^b^	(112.4± 0.2)^c^	(59.3± 1.2)^a^	(11.3± 0.5)^a^	(21.9± 3.0)^a^	(1.16± 0.17)^b^	(5.3± 0.2)^a^	(3.2± 0.0)^a^	(30.1± 0.1)^b^

*E*=total energy, GAE=gallic acid equivalents, TAA=total antioxidant activity, TE=Trolox equivalents, *v*=specific volume, WA=water absorption. The results are shown as mean value±standard deviation of duplicate analyses. Values in the same column with different letters differ significantly (p<0.05)

No significant differences (p>0.05) were detected in the total ash and moisture mass fractions in different biscuit samples (Table 3). On the other hand, the fat mass fraction in the biscuits was mainly affected by the addition of chia seeds, and the biscuits with chia seeds had significantly higher mass fractions of fat (p<0.05). Increase in the total dietary fibre and fat mass fractions in biscuit samples with chia seeds were also evident in previous study (*42*). Previous findings on the nutritional properties of gluten-free products that combine chia seeds and buckwheat flour highlighted that these products had health-promoting benefits for obese and diabetic individuals by lowering their glycaemic index, increasing satiety, in addition to being gluten-free (*44*). Among all biscuit samples, the dietary fibre mass fraction was the lowest in biscuits with buckwheat (p<0.05). This finding was in parallel with low mass fraction of dietary fibre in buckwheat flour.

### Total phenolic content in biscuit samples

The use of ingredients with relatively high amount of phenolic compounds in gluten-free products has a significant benefit for coeliac patients due to their ability to bind to fibre moieties, thus making them readily absorbable in the colon. In addition to the direct antioxidant activity of phenolic compounds, this binding ability was proposed as a beneficial mechanism for coeliac patients by modifying the composition and immune function of the gut microbiota (*45*).

The total phenolic content (expressed as gallic acid equivalents (GAE) on dry mass basis) of the biscuit samples changed from 248.2 to 400.2 mg/100 g (Table 3). Biscuits with chestnut had the highest phenolic content among all samples (400.2 mg/100 g). Biscuits with buckwheat also contained relatively high amounts of phenolics (387.0 mg/100 g). A previous review on chestnuts reports high but varying contents of total phenolics (mainly as gallic and ellagic acid) in chestnut fruits (280–910 and 610–2560 mg/100 g) (*46*).

Chia seed supplementation to the biscuit formulation, on the other hand, significantly (p<0.05) decreased the total phenolics (Table 3). The amount of chia seed incorporation may affect the total phenolic content. In a previous research, total phenolic content was 156.99 µg/g in cooked durum wheat pasta, but lower when 5% chia seeds were added into samples (123.53 µg/g) and higher when 10% chia seeds were added into samples (186.80 µg/g) (*8*). In addition, the relative decrease in total phenolic content after chia seed supplementation may be related to the richness of chia seeds not only in the nutritional compounds but also in the non-nutritional healthy compounds (*44*).

### Total antioxidant activity of biscuit samples

Total antioxidant activity (TAA), expressed as Trolox equivalents (TE) on dry mass basis, of the biscuits varied between 78.2 and 155.5 mg/100 g (Table 3). In parallel with total phenolics, biscuits with chestnut had the highest TAA and the addition of chia seeds significantly reduced the measured TAA. In the literature, increases in the measured level of antioxidant activity after baking the biscuits containing buckwheat flour or chestnut flour were also observed and this was explained as the effect of Maillard reaction products formed during heat treatment (*2*, *47*). Relatively low antioxidant activity of chia seeds was also reported and related to the presence of hydrophilic phenolic acids in the seeds (*48*).

### Physical properties of biscuits

Table 3 shows the quality parameters of gluten-free biscuits. Although no significant differences were detected in the physical parameters of diameter, height and mass of the biscuit samples (p>0.05), chia reduced the volume of the biscuits slightly. This tendency to decrease specific volumes was also evident in bread samples with chia seeds and in a previous study, protein network interruption was presented as the main reason for this change (*49*). However, the gluten network development in biscuits is quite limited because of its high fat and sugar contents. Therefore, the disruption of gluten network is not as critical in biscuits as it is in bread, and the effect of chia seeds on the volume is rather restricted in the samples presented in this study (*9*).

Spread ratio (diameter/thickness ratio) is among the most important characteristics of biscuits in determining their quality. Spread ratio values were higher (p>0.05) in the biscuits with buckwheat flour than in its chestnut flour-containing counterparts (Table 3). Moreover, chia seeds seemed to cause a slightly increasing effect on spread ratio. In the previous studies, the spread ratio was strongly linked to the chemical composition of the raw materials in addition to the interference of sugar and fat and relatively low dough viscosity (*14*).

#### Hardness

Hardness of biscuits is among the distinctive textural parameters for consumer acceptability and general textural properties of biscuits are highly related to the starch gelatinization and sugar content, rather than the protein/starch structure (*9*). Hardness ranged between 30.1 and 48.7 N, with the statistically significant (p<0.05) difference between the buckwheat and the other samples (Table 3). Effect of buckwheat flour on the increased hardness of bakery products was also presented in the literature (*13*, *31*). Buckwheat flour substitution was reported to increase the hardness (from 24.63 to 42.30 N) in gluten-free biscuits, in comparison with the wheat flour samples (*36*). Increased hardness was also presented as undesirable during the shelf-life of buckwheat flour-containing gluten-free biscuits (*14*). In the previous study, the presence of sucrose in chestnut flour was mentioned as a factor that affects the rheological properties of bakery products by inhibiting the hydration of starch granules and starch gelatinization (*37*). However, its effect was not as precise as the hardness of buckwheat samples. Decrease in the hardness with the addition of chia seeds might be related to the ability of chia seeds to absorb more water (*36*).

According to the results in Table 3, the highest water absorption was measured in the biscuits with buckwheat flour and chia (3.3 g). The use of chia seeds with either buckwheat or chestnut flour increased the measured amount of water absorption. Literature reports on the increase in the water absorption of gluten-free biscuits containing buckwheat flour and gum (*36*). The extent of water absorption is related to the interaction of water and the ingredients, the number of hydration positions and protein configuration. In this context, the presence of more protein and fibre in chia seed gel was suggested as the origin of the relatively higher amount of water (*50*).

#### Colour

**Fig. 1:**
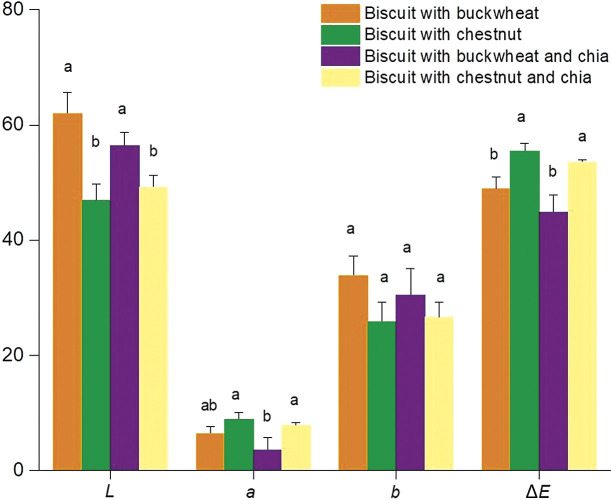
Colour values of biscuit samples. The results are shown as mean value±standard deviation. Different letters in column for each parameter show that the results differ significantly (p<0.05)

Biscuit colour is mostly related to the colour of the ingredients, in addition to the browning during the advanced stage of the Maillard reaction and caramelization. Colour parameters of darkness/lightness (L* values), redness (a* values) and yellowness (b* values) in addition to total colour change (Δ*E*) were determined and the results are given in Fig. 1. Samples with buckwheat flour were lighter, having significantly higher L* values than the biscuits with chestnut flour (p<0.05). Addition of chia seeds did not seem to make any significant changes (p>0.05) in the lightness. In the biscuits with chestnut flour, redness values were slightly higher (higher a* values), while the addition of chia seeds had a lowering effect on redness. Although yellowness was slightly higher in the samples with buckwheat flour, the difference was not statistically significant (p>0.05). Δ*E* data revealed that chestnut flour had a higher effect on the colour of biscuits (p<0.05). Similar to our findings, the use of chestnut flour as a substitute for wheat flour (increasing from 20 to 60% and 0 to 100%) resulted in the increase of the redness of the cookies and bread samples, respectively (*37*, *51*). In the literature, chestnut flour with its relatively high content of sugar (20-32%) and starch (50-60%) was related with the caramelization and Maillard reactions due to baking (*2*). These reactions were characterized by the decreased L* values and increasing a* values in the samples.

### Sensorial properties of the biscuits

**Fig. 2:**
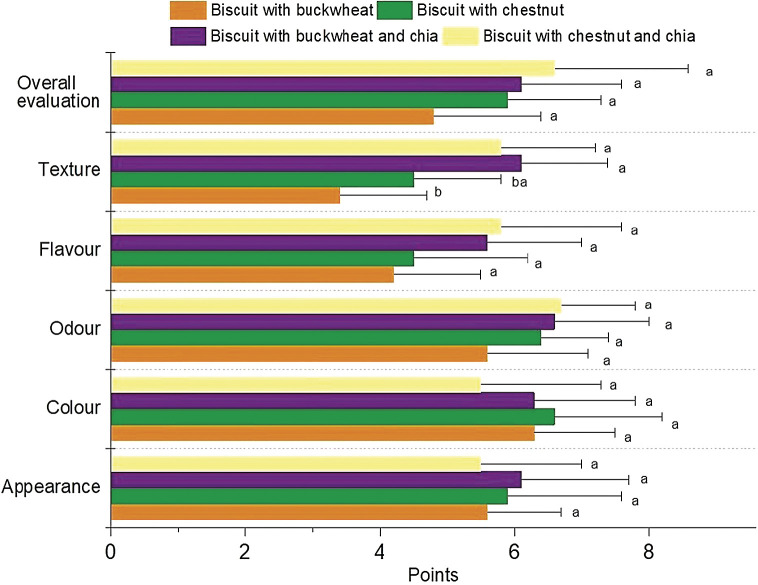
Sensory scores of biscuit samples. The results are presented as mean value±standard deviation. Different letters in column for each parameter show that the results differ significantly (p<0.05)

The results of the sensory evaluation are shown in Fig. 2. According to the sensory evaluation results, only texture was indicated as significantly different among the biscuit samples, as the addition of chia seeds significantly increased the scores for the texture (p<0.05). The overall evaluation of the panellists illustrated a moderately higher acceptance (p>0.05) of biscuits supplemented with chia seeds (both buckwheat and chestnut flour-based biscuits) and the biscuits formulated with buckwheat flour and chia received the highest scores for appearance, flavour and texture.

Biscuits with buckwheat generally had the lowest results (except for the colour and appearance) in terms of the evaluated sensory parameters. Studies indicating that the level of buckwheat flour substitution in biscuits affected their sensorial acceptance could be found (*52*), where the biscuits with 40% of flour substituted with buckwheat flour had the highest score among three levels (30, 40 and 50%) of substitution, while the overall acceptability was measured in the samples with added xanthan gum. The presence of some phenolic compounds such as rutin, quercetin and protocatechuic acid in buckwheat flour was represented as the main factor for lower scores of buckwheat-incorporated products (*52*).

In the literature, lower scores in sensory evaluation of buckwheat-incorporated gluten-free biscuits were improved by adding gum acacia and guar gum into the formulations (*15*). Increase in sensory scores was explained by different interactions occurring between the hydrocolloids and other food ingredients that resulted in improved textural properties. Comparable increasing scores for flavour and overall acceptance after the addition of chia seeds into chestnut biscuits were also evident. This finding is similar to previous findings as combinations of chestnut flour and rice flour with different gums and emulsifiers provided increased acceptance scores in sensory evaluation of the bread samples (*15*).

Visual properties of appearance and colour of biscuits with chestnut flour were ranked higher than the biscuits with buckwheat flour (Fig. 2). Positive contribution of chestnut flour to the properties such as appearance, shape, crumb structure, *etc*. was in agreement with the previous studies (*37*). More specifically, consumer preference for darker colour in gluten-free bakery products was highlighted in previous studies (*19*).

Although the flavour scores were not significantly different among different formulations (p>0.05), chia seeds seemed to positively affect the flavour of buckwheat biscuits (Fig. 2). Due to the perceived bitterness, the buckwheat flour was reported to affect the product flavour adversely, particularly at high (40%) substitution levels (*10*).

### Influence of storage time on biscuits

#### Physicochemical changes

According to the results in Table 4, no statistically significant difference was found among the initial moisture mass fractions of the biscuit samples (as of day 1) (p>0.05). Initial moisture mass fraction measured in this study ranged between 5.2% (the lowest for the biscuits with buckwheat flour) and 5.9% (the highest for the biscuits with chestnut flour). During the investigated storage period, all the samples tended to absorb moisture and significant increases were evident as of the 20th day of storage (p<0.05) in each sample (Table 4). On the other hand, a milder increase was evident after the 20th day, as no more significant changes were measured from the 20th to the 45th day of storage (p>0.05). During storage, the biscuits with chestnut flour absorbed more moisture than the biscuits with buckwheat (p<0.05). This finding was in accordance with the literature (*2*), indicating that biscuit samples containing chestnut flour had increased moisture content as the storage period progressed, and particularly significant increases in moisture absorption were detected on the 30th and 60th days of storage. Chia seeds had no effect on the moisture content of the biscuits.

**Table 4:** Changes of moisture, pH and peroxide value during storage of biscuits

Sample	*t*(storage)/day								
1			20			45		
*w*(moisture)/ %	pH	PV(as O_2_)/ (mmol /kg)	*w *(moisture)/ %	pH	PV(as O_2_)/ (mmol /kg)	*w*(moisture)/ %	pH	PV(as O_2_)/(mmol/kg)
Biscuit with buckwheat	(5.2± 0.3)^ay^	(5.57± 0.01)^ax^	(1.08± 0.01)^cy^	(8.3± 1.0)^bx^	(5.40± 0.00)^by^	(1.14± 0.01)^cy^	(8.8± 0.3)^bx^	(5.27± 0.01)^bz^	(1.31± 0.11)^cx^
Biscuit with chestnut	(5.9± 0.3)^ay^	(5.62± 0.02)^ax^	(1.55± 0.04)^ay^	(11.0± 1.0)^ax^	(5.60± 0.01)^ax^	(1.72± 0.16)^ax^	(11.8± 0.2)^ax^	(5.55± 0.01)^ay^	(1.81± 0.03)^bx^
Biscuit with buckwheat and chia	(5.4± 0.8)^ay^	(5.18± 0.03)^bx^	(1.11± 0.05)^cy^	(8.9± 0.5)^abx^	(5.17± 0.01)^dx^	(1.22± 0.04)^cy^	(9.1± 0.4)^bx^	(5.21± 0.01)^cx^	(1.4± 0.2)^cx^
Biscuit with chestnut and chia	(5.5± 0.5)^ay^	(4.96± 0.04)^cy^	(1.30± 0.06)^bz^	(10.7± 0.4)^ax^	(5.23± 0.01)^cx^	(1.50± 0.06)^by^	(11.2± 0.7)^ax^	(5.28± 0.01)^bx^	(1.95± 0.03)^ax^

The results are shown as mean value±standard deviation of duplicate analyses. Moisture content, pH and peroxide value (PV) with different letters (a-c) within the same column and different letters (x-z) within the same row differ significantly (p<0.05)

Change in pH during storage might affect the perception of the flavour of biscuits and values near neutral pH are more preferred. According to the results, pH of the samples varied between 4.96 and 5.62 as of day 1 (Table 4). Samples with chia seeds had significantly lower pH (p<0.05). Changes of pH of each sample during storage differed significantly. The pH values decreased significantly (p<0.05) in biscuits with buckwheat flour (5.55 to 5.27) and biscuits with chestnut flour (5.62 to 5.55) during storage, and this was in accordance with the previous findings, as the incorporation of barley, gram, millet and maize flour similarly decreased the pH of the biscuits during ambient storage for 60 days (*53*). The pH of both biscuits with chia seeds fluctuated during storage, although the only significant increase was evident in the biscuits with chestnut flour and chia seeds from day 1 to 20. These findings were different from those of Mesias *et al.* (*42*), who found that chia seed addition (5-20%) into wheat flour resulted in a decreasing trend in pH from 7.6 to 7.3.

#### Peroxide value

Peroxide value (PV) is the most common indicator for measuring the oxidation during storage. Significant increase of PV values during storage was detected in all samples (p<0.05). Oxidation stability of biscuits with chestnut flour and chia seeds was found to be lower than the remaining samples at the end of storage, with relatively high PV expressed as O_2_ (1.95 mmol/kg). This is expected, since the total amount of chia seeds in the biscuit formulation is around 10% of the total flour content, with mostly unsaturated fatty acids. In the literature, the PV of cookies with added chia seeds (10%) was reported as 2.81 mmol/kg at the end of 30 days of storage (*54*).

#### Fatty acid composition

The fatty acid composition of the biscuit samples (after the 1st day and after 6 months of storage) is presented in Table 5. Initially, only the caprylic, palmitic and margaric acid mass fraction differed significantly among different biscuits (p<0.05). Palmitic, oleic, linoleic, lauric and stearic acids were the dominant fatty acids in the produced biscuit samples. Throughout the shelf life, the losses in lauric, stearic and α-linolenic acids were evident (p<0.05). Other fatty acids detected in biscuit samples were relatively stable during the period in which they were stored. The use of margarine in the biscuit formulations and the presence of antioxidants from buckwheat and chestnut flour might have contributed to this stability (*2*). Chia seeds have a high amount of total fat, mostly rich in α-linolenic acid (ω3) and also linoleic acid (ω6). In the literature, different colours of chia seeds were detected with no difference in their fatty acid profiles (*55*). According to the findings, palmitic, stearic, oleic, linoleic and α-linolenic fatty acids were detected in both white and black-spotted chia seeds from five different locations, in addition to trace amounts of myristic, arachidic, gadoleic, behenic, erucic and lignoceric acids. However, the total amount of oil in chia seeds and palmitic, oleic, linoleic and α-linolenic fatty acids among oils from different locations were significantly different (*55*). Therefore, the relatively low amount of α-linolenic acid (ω3) might be related to the type of chia seeds used in the formulations in this study.

**Table 5:** Fatty acid composition of the biscuit samples

Fatty acid	*t*(storage)=1 day				*t*(storage)=6 months				
Biscuit with buckwheat	Biscuit with chestnut	Biscuit with buckwheat and chia	Biscuit with chestnut and chia	Biscuit with buckwheat	Biscuit with chestnut		Biscuit with buckwheat and chia	Biscuit with chestnut and chia
*w*(fatty acid)/%								
Butyric (C4:0)	(0.24±0.01)^A^	(0.11±0.02)^A^	(0.11±0.02)^A^	(0.24±0.09)^A^	(0.18±0.08)^a^ -		(0.3±0.1)^a^ -	(0.17±0.04)^a^ -	(0.3±0.1)^a^ -
Caprylic (C8:0)	(2.72±0.04)^A^	(1.27±0.08)^B^	(1.6±0.2)^B^	(3.0±0.5)^A^	(1.5±0.6)^a^ -		(1.7±0.8)^a^ -	(1.3±0.2)^a^ #	(2.0±0.9)^a^ -
Capric (C10:0)	(176±0.2)^A^	(1.11±0.00)^A^	(1.3±0.1)^A^	(2.0±0.6)^A^	(1.1±0.4)^a^ -		(1.3±0.6)^a^ -	(0.97±0.06)^a^ -	(1.4±0.7)^a^ -
Lauric (C12:0)	(14.3±2.3)^A^	(12.7±0.2)^A^	(13.6±1.3)^A^	(17.5±5.2)^A^	(9.8±2.1)^a^ #		(10.9±4.9)^a^ -	(8.9±2.3)^a^ -	(11.02±5.66)^a^ -
Myristic (C14:0)	(4.2±0.3)^A^	(4.4±0.1)^A^	(4.2±0.5)^A^	(4.7±1.0)^A^	(3.5±0.2)^a^ -		(3.8±1.0)^a^ -	(3.4±0.8)^a^ -	(3.8±1.8)^a^ -
Palmitic (C16:0)	(28.7±0.7)^AB^	(30.3±0.4)^A^	(29.2±1.2)^AB^	(27.78±0.07)^B^	(31.4±1.5)^a^ -		(29.3±0.2)^a^ -	(29.6±1.7)^a^ -	(29.8±1.0)^a^ -
Palmitoleic (C16:1)	(0.24±0.00)^A^	(0.29±0.01)^A^	(0.30±0.08)^A^	(0.26±0.01)^A^	(0.23±0.02)^a^ -		(0.19±0.00)^a^ #	(0.17±0.01)^a^ -	(0.3±0.1)^a^ -
Margaric (C17:0)	(0.08±0.00)^B^	(0.09±0.00)^B^	(0.08±0.00)^B^	(0.44±0.05)^A^	(0.08±0.01)^a^ -		(0.09±0.01)^a^ -	(0.08±0.01)^a^ -	(0.10±0.03)^a^ #
Stearic (C18:0)	(10.2±0.4)^A^	(10.0±0.2)^A^	(10.7±0.9)^A^	(9.0±1.8)^A^	(7.1±0.2)^a^ #		(7.9±1.1)^a^ -	(8.2±0.8)^a^ -	(8.0±0.2)^a^ -
Elaidic (C18:1)	(0.09±0.00)^A^	(0.10±0.00)^A^	(0.13±0.02)^A^	(0.10±0.02)^A^	(0.07±0.01)^a^ -		(0.12±0.04)^a^ -	(0.10±0.01)^a^ -	nd -
Oleic (C18:1)	(21.0±1.0)^A^	(21.8±0.3)^A^	(21.6±1.5)^A^	(19.5±3.3)^A^	(28.1±1.0)^a^ *		(31.1±4.2)^b^ -	(32.4±2.7)^a^ -	(29.49±0.00)^a^ -
Linoleic (ω6; C18:2)	(14.7±0.6)^A^	(15.7±0.2)^A^	(15.2±1.0)^A^	(16.1±2.3)^A^	(15.2±0.6)^b^ -		(11.3±1.5)^c^ -	(12.4±1.0)^bc^ -	(19.7±1.4)^a^
α-Linolenic (ω3; C18:3)	(1.31±0.11)^A^	(1.50±0.02)^A^	(1.5±0.1)^A^	(1.5±0.3)^A^	(0.73±0.06)^b^ #		(1.0±0.2)^ab^ -	(1.2±0.1)^a^ -	(1.06±0.07)^a^ -
Arachidic (C20:0)	(0.31±0.02)^A^	(0.29±0.00)^A^	(0.35±0.02)^A^	(0.3±0.1)^A^	(0.38±0.01)^a^ *		(0.40±0.08) ^a^ -	(0.43±0.07)^a^ -	(0.36±0.06)^a^ -

The results are shown as mean value±standard deviation of duplicate analyses. Values with different letters in the same rows for the 1st day of storage (A-B) and 6 months of storage (a-c) differ significantly (p<0.05). The insignificant difference (p>0.05) between the 1st day and 6 months of storage is represented by **-**, the significant increase (p<0.05) by * and significant decrease (p<0.05) by #

## CONCLUSIONS

Recent research on gluten-free products has led to the improvement of nutritional profile of these products as the primary point of interest. The results presented here confirm the importance of using functional ingredients in gluten-free biscuit formulations as well as monitoring their storage stability. The results confirm the relative stability of biscuits during storage, particularly of those containing chestnut flour and chia seeds. Therefore, it was concluded that these functional ingredients have the potential to be used in different gluten-free product formulations (biscuits, crackers, snacks, *etc*.), with significant protein and dietary fibre contents, superior product quality and better sensory acceptability.
